# Tumor neutrophils ferroptosis: a targetable immunosuppressive mechanism for cancer immunotherapy

**DOI:** 10.1038/s41392-023-01357-z

**Published:** 2023-02-22

**Authors:** Songtao Du, Furong Zeng, Guangtong Deng

**Affiliations:** 1 Department of Dermatology, Xiangya Hospital, Central South University, Changsha, China; 2 Department of Colorectal Surgical Oncology, The Tumor Hospital of Harbin Medical University, Harbin, China; 3 Department of Oncology, Xiangya Hospital, Central South University, Changsha, China

**Keywords:** Cancer microenvironment, Tumour immunology

In a recent study published in Nature, Kim et al. demonstrated that ferroptosis of polymorphonuclear myeloid-derived suppressor cells (PMN-MDSCs) causes immune suppression in cancer,^1^ highlighting ferroptosis inhibition as a targetable therapeutic strategy for cancer immunotherapy.

**Fig. 1:**
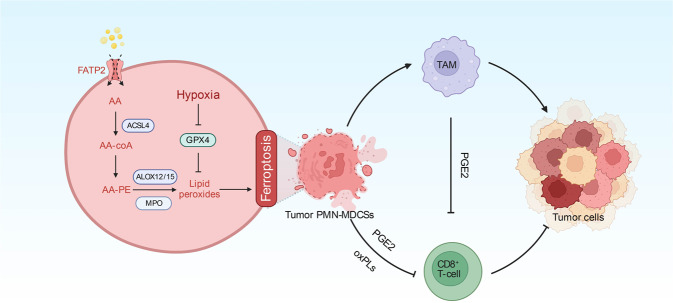
Ferroptosis of PMN-MDSCs causes immune suppression in cancer. Spontaneous ferroptosis of PMN-MDSCs in the tumor microenvironment triggers the secretion of PGE2 and the release of oxidized phospholipids, rendering them more immunosuppressive through influencing the activity of CD8^+^ T cells and tumor-associated macrophages. AA arachidonic acid, ACSL4 acyl-coenzyme A synthetase long-chain family member 4, Alox12/15 arachidonate 12/15-lipoxygenase, GPX4 glutathione peroxidase 4, PE phosphatidylethanolamine, MPO myeloperoxidase, PGE2 prostaglandin E2, PMN-MDSCs pathologically activated neutrophils termed myeloid-derived suppressor cells, TAM tumor-associated macrophage. The images were created with BioRender.com

Ferroptosis, a regulated cell death characterized by iron-dependent accumulation of polyunsaturated fatty acid peroxides, has been regarded as a potent antitumor approach. However, most of the studies rely primarily on xenograft mouse tumor models, which lack a functional immune system. Thus, the effect of ferroptosis on immune cells, especially tumor PMN-MDSCs, is poorly understood. Interestingly, the ferroptosis pathway was found to be enriched in PMN-MDSCs in colorectal cancer liver metastasis using single-cell transcriptome analysis.^2^ Kim et al. further reported that the spontaneous ferroptosis of PMN-MDSCs in the tumor microenvironment triggered the secretion of PGE2 and the release of oxidized phospholipids, rendering them more immunosuppressive by influencing the activity of CD8^+^ T cells and tumor-associated macrophages (Fig. 1).

First, the authors found that several genes involved in ferroptosis, as well as the ferroptosis gene signature, were upregulated in tumor PMN-MDSCs, compared with PMN-MDSCs from matched and peripheral blood of healthy donors. The authors then measured oxidized phosphatidylethanolamine-containing arachidonic acid (AA-PEox), the hallmark of ferroptosis, using mass spectrometry, to find increased accumulation of AA-PEox in tumor PMN-MDSCs. The tumor PMN-MDSCs were more sensitive to ferroptosis inducers, and spontaneous death could be rescued by not only inhibitors of apoptosis, but also inhibitors of ferroptosis. Moreover, the levels of lipid-derived signals associated with ferroptosis and CD71, a well-known ferroptosis biomarker, were highly expressed in tumor PMN-MDSCs. These results indicate that tumor PMN-MDSCs are susceptible to ferroptosis and even undergo ferroptosis spontaneously.

Second, to investigate the association between ferroptosis and immunosuppressive function in tumor PMN-MDSCs, the authors treated non-suppressive PMNs with ferroptosis and apoptosis inducers. As measured by the reduction in CD8^+^ T cell proliferation, only ferroptosis inducers could achieve immunosuppressive activity, with this suppressive effect being mediated by soluble factors. Genetic inhibition of ferroptosis by *Alox12/15* deletion or *Acls4* deletion, or inhibitors of ferroptosis, erased the immunosuppressive effect of tumor PMN-MDSCs in different murine models and patient samples. Gene set enrichment analysis suggested that the blockade of ferroptosis by *Alox12/15* deletion in tumor PMN-MDSCs caused the upregulation of genes associated with the classical activation of PMNs, including complement activation, neutrophil-mediated immunity, monocyte chemotaxis, and antigen processing and presentation. These results suggest that ferroptosis mediates the conversion of classical PMNs to PMN-MDSCs, but the mechanism requires further investigation.

Third, the authors identified two kinds of soluble factors that regulate the ferroptosis-mediated immune suppression of PMN-MDSCs, prostaglandin E2 (PGE2) and oxidized phospholipids. Consistent with the upregulation of genes involved in the biosynthesis of PGE2 after RSL3 (a ferroptosis-inducer) treatment, the production of PGE2 was substantially increased after ferroptosis induction in tumor PMN-MDSCs. However, neither blockers of PGE2 synthetic enzymes (cyclooxygenase-1 and cyclooxygenase-2), nor inhibitors of PGE2 receptors (EP2 and EP4) completely erased the suppressive effect of ferroptosis induction. Oxidized phospholipids, the major products of ferroptosis, can directly cause the inhibition of CD8^+^ T cell proliferation. These results suggest that the release of oxidized phospholipids can also contribute to the ferroptosis-mediated suppression of PMN-MDSCs, in addition to the secretion of PGE2. Unsurprisingly, other factors that may promote polyunsaturated fatty acid phospholipid (PUFA-PL) synthesis and peroxidation (such as FATP2 and MPO), or that may cause GPX4 inhibition (such as hypoxia in the tumor microenvironment), may also influence the inhibitory activity of tumor PMN-MDSCs. Arachidonic acid, in combination with T cell-derived IFNγ, has been reported to cause immunogenic tumor ferroptosis.^3^ It is unclear whether other secreted factors such as free fatty acids could promote tumor PMN-MDSC ferroptosis.

Finally, the authors investigated the effect of pharmacological inhibition of ferroptosis on xenograft mouse tumor models. Unexpectedly, liproxstatin-1 treatment did not reduce tumor growth when treatment was initiated when tumors were approximately 100 mm^3^, although suppressive activity of tumor PMN-MDSCs was substantially inhibited. However, tumor growth was markedly inhibited in immunocompetent (but not immunodeficient) mice when using liproxstatin-1 at an earlier time when tumors were palpable. Flow cytometric analyses showed that liproxstatin-1 treatment increased the proportion of resident memory, central memory, effector CD8^+^ T cells, and natural killers in tumor microenvironment. Moreover, T cell activation was observed by scRNA-seq, characterized by upregulation of IL-2 and TNFα signaling and inflammatory response, and downregulation of oxidative phosphorylation. These results indicate that T cell infiltrating and activation contribute to the tumor regression with liproxstatin-1 treatment. The authors also observed a synergism of liproxstatin-1 treatment with PD-1 blockade. Additionally, analysis of patient data from The Cancer Genome Atlas showed strong correlations between tumor PMN-MDSCs and ferroptosis signatures in multiple cancer types. High expression of the ferroptosis signature was associated with lower survival. The ferroptosis signature was positively associated with tumor immunogenicity, and ferroptosis inducers could inhibit tumor growth and sensitize the tumors to IFNγ and immune checkpoint blockade.^4^ Moreover, ferroptosis in tumor-infiltrating CD8^+^ T cells could impair anti-tumor immunity and promote tumor progression.^5^ Thus, ferroptosis induction for cancer therapy must consider the complex nature of ferroptosis in the tumor microenvironment.

In summary, the present findings not only reveal a previously unrecognized role of ferroptosis-mediated immune suppression in PMN-MDSCs, but also raise a paradox in the use of ferroptosis inducers in tumor therapy. However, the ferroptosis inducers or inhibitors used in the study were not cell-specific, which could affect the viability and activity of other cells in the tumor microenvironment and finally cause the bias and misinterpretation of results. Thus, cell-specific delivery of ferroptosis inducers or inhibitors presents a promising and contentious area for future research. Like any pioneering study, such an ingenious study also promotes additional, interesting questions for the future. Why does the immunosuppressive effect of ferroptosis in tumor PMN-MDSCs outweigh the tumor-killing effect of ferroptosis in tumor cells? How does ferroptosis in tumor PMN-MDSCs affect other immune cells in addition to tumor-infiltrating CD8^+^ T cells and tumor-associated macrophages? What is the role of ferroptosis for other immune cells such as dendritic cells and Treg cells in the tumor microenvironment?
